# Supplementary material for: Disruption of myelin structure and oligodendrocyte maturation in a macaque model of congenital Zika infection
Source: Nat Commun. 2024 Jun 18;15:5173. doi: 10.1038/s41467-024-49524-2 (PMC11189406; doi:10.1038/s41467-024-49524-2)
Supplement: Supplementary file 1 — Supplementary Information [file 41467_2024_49524_MOESM1_ESM.pdf]

SUPPLEMENTAL INFORMATION

Disruption of myelin structure and oligodendrocyte maturation in a macaque model of congenital Zika infection

Table of Contents

Tables ……………………………………………………………………………………………… Page

Table S1. Species, Sex, Age and Gestational Days of the Maternal Animals 2

Table S2. Species, Sex, Age and Weights of the Fetuses 3

Table S3. Primary antibodies used for IHC and DSP analyses 4

Supplemental Figures……………………………………………..…………................ Page

Figure S1. Maternal ZikV infection study design and NHP fetal brain sampling overview 5-6

Figure S2. Spatial transcriptomic analysis of NHP fetal brain responses to ZikV infection in discrete brain regions 7-8

Figure S3. Global RNA sequencing of ZikV and control NHP fetal superficial cortex 9-10

Figure S4. Immunohistological characterization of ZikV and control NHP fetal brains 11-12

Figure S5. Histopathologic changes in the periventricular region of the occipital cortex lesion of ZikV and control NHP fetal brain 13-14

Figure S6. Neuron density by cortical layer in parietal and occipital cortex of ZikV and control NHP fetal brains 15

Figure S7. Electron microscopy analysis of axon and myelin features in deep white matter of ZikV and control NHP fetal brains 16-17

Figure S8. Graphical abstract representing putative mechanisms for ZikV-induced myelin decompaction, including altered oligodendrocyte maturation, and neuronal function 18

Table S1. Species, Sex, Age and Gestational Days of the Maternal Animals

Study ID	Animal ID	Species	Sex	Age (y)	Gestational Age		Days post-inoculation
					Inoculation	Delivery	
ZIKA1	A10095	*M. nemestrina*	F	9.5	119	158	39
ZIKA2	F10094a	*M. nemestrina*	F	5.9	82	159	77
ZIKA3*	A10219a	*M. nemestrina*	F	10.0	63	150	87
ZIKA4*	T04054	*M. nemestrina*	F	12.8	60	142	82
ZIKA5*	J07322	*M. nemestrina*	F	9.1	60	157	97
ZIKA6	A10008a	*M. nemestrina*	F	12.2	118	141	23
CTL1	A07083	*M. nemestrina*	F	14.0	59	159	100
CTL2	M05062	*M. nemestrina*	F	12.0	63	155	92
CTL3	F09165a	*M. nemestrina*	F	7.4	99	156	57
CTL4	A10183a	*M. nemestrina*	F	7.8	N/A	156	N/A
CTL5	Z15006	*M. nemestrina*	F	5.2	138	158	20
CTL6	A10007	*M. nemestrina*	F	11.0	132	155	23

Abbreviations: F, Female; y, years (date of departure)

*Animals that received a mosquito salivary preparation inoculation and administration of DENV Ab, as previously described{Adams Waldorf, 2018 #31}.

ZIKA refers to animal experiments with ZikV subcutaneous inoculation.

CTL, refers to animal experiments with media subcutaneous inoculation that underwent similar procedures as the ZikV animals.

N/A, not applicable

The average gestational age at delivery for pigtail macaques in the WaNPRC colony is 172 days gestation.

All animals were delivered by C-Section in the absence of labor.

Table S2. Species, Sex, Age and Weights of the Fetuses

Study ID	Animal ID	Species	Sex	Age (d)	Body weight (kg)	Brain weight (kg)	Biparietal diameter (mm) / gestational age (day) at measurement	White matter fraction of supratentorial volume / gestational age (day) at measurement
ZIKA1	Z16128	*Macaca nemestrina*	M	158	0.453	N/A	54.8 / 158	0.55 / 129
ZIKA2	Z16216	*Macaca nemestrina*	F	159	0.451	N/A	52.2 / 158	0.56 / 124
ZIKA3	Z16351	*Macaca nemestrina*	F	150	0.385	0.0514	47.9 / 149	0.56 / 120
ZIKA4	Z16354	*Macaca nemestrina*	F	142	N/A	N/A	44.9 / 121	0.54 / 121
ZIKA5	Z17003	*Macaca nemestrina*	F	157	0.453	0.0594	53.3 / 156	0.54 / 120
ZIKA6	Z19249	*Macaca nemestrina*	F	141	0.317	0.0472	N/A	N/A
CTL1	Z16296	*Macaca nemestrina*	F	159	0.432	0.0578	51.6 / 157	0.58 / 124
CTL2	Z17059	*Macaca nemestrina*	F	155	0.467	0.0563	51.7 / 153	0.57 / 118
CTL3	Z17004	*Macaca nemestrina*	F	156	0.352	N/A	50.0 / 155	0.59 / 125
CTL4	N/A	*Macaca nemestrina*	F	156	N/A	N/A	N/A	N/A
CTL5	Z20054	*Macaca nemestrina*	F	158	0.499	0.0559	N/A	N/A
CTL6	Z21105	*Macaca nemestrina*	M	155	0.573	0.0601	N/A	N/A

Abbreviations: F, Female; M, Male; d, days; kg, kilogram
ZIKA, refers to animal experiments with ZikV subcutaneous inoculation.
CTL, refers to animal experiments with media subcutaneous inoculation that underwent similar procedures as the ZikV animals.
N/A, not available (not measured)
Biparietal diameter and white matter fraction were calculated from the last MRI time point prior to necropsy.

Table S3. Primary antibodies used for IHC and DSP GeoMx analyses

Antibody	Source (cat #)	Species	Clone	Assay	Target	Dilution
GFAP	Dako (Z0224)	rabbit polyclonal		IHC	astrocytes	1:500
AIF-1/Iba1	Novus Biologicals, (MBP2-19019)	rabbit polyclonal		IHC	microglia	1:500
Olig2	Millipore (AB9610)	rabbit polyclonal		IHC	oligodendrocytes	1:500
NeuN	Millipore (MAB377)	mouse monoclonal	Clone A60	IHC	neurons	1:500
MBP	Abcam (ab7349)	rat monoclonal	Clone 12	IHC	myelin sheath	1:500
RBFOX3 (NeuN)	Abcam (ab190195)	monoclonal	EPR12763	DSP	neurons	1:50
Olig2	Millipore (AB9610)	rabbit polyclonal		DSP	oligodendrocytes	1:100
GFAP	Novus Biologicals (NBP-33184DL594)	mouse monoclonal	Clone GA-5	DSP	astrocytes	1:400
STYO 83	ThermoFisher (S11364)			DSP	nuclei	
Goat anti-Rb AF647	ThermoFisher (A27040)	Goat	Superclonal	DSP	Rabbit IgG	

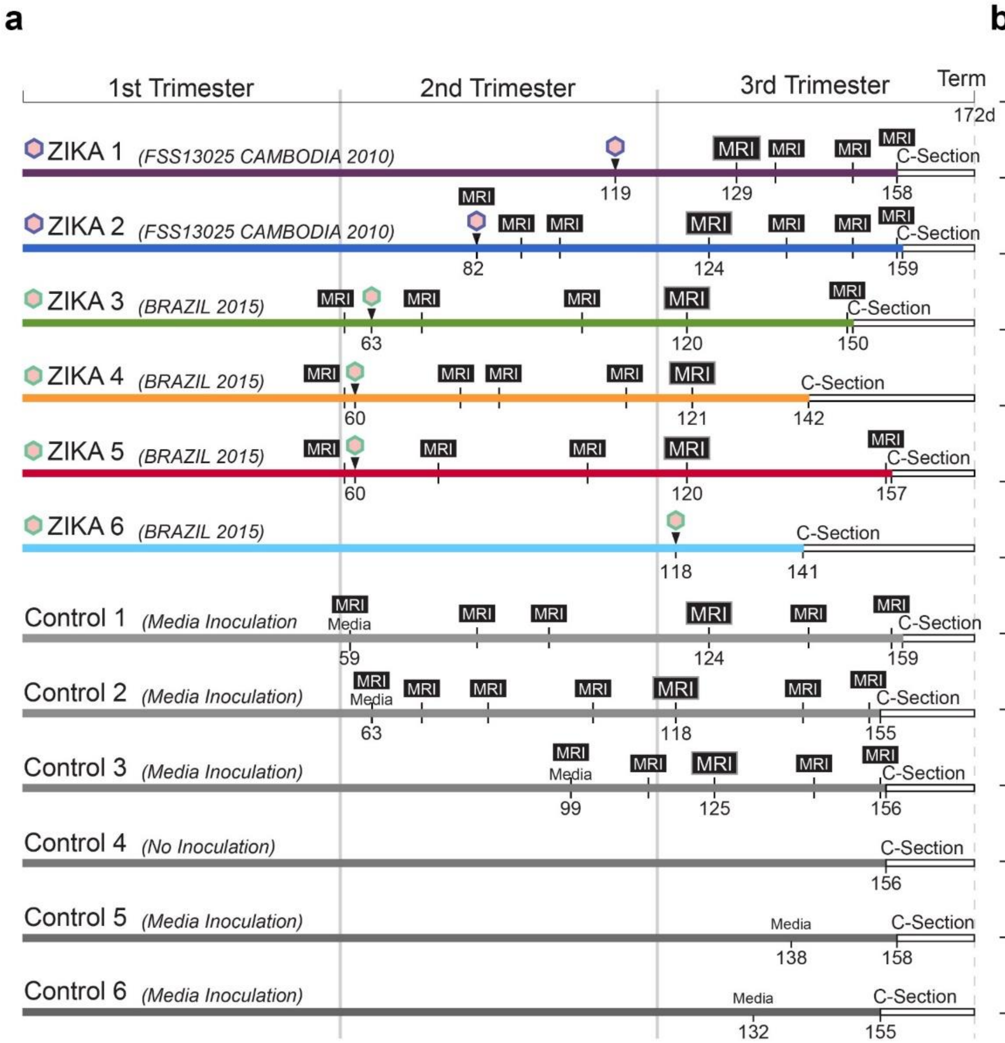

b

Animal	DSP	RNAseq	IHC/H&E	MRI	EM
Z1	X	X	X	X	
Z2		X	X	X	
Z3	X	X	X	X	X
Z4	X	X	X	X	
Z5	X	X	X	X	X
Z6	X		X		X
C1	X	X	X	X	X
C2	X	X	X	X	X
C3	X	X	X	X	X
C4	X		X		
C5	X		X		X
C6	X		X		

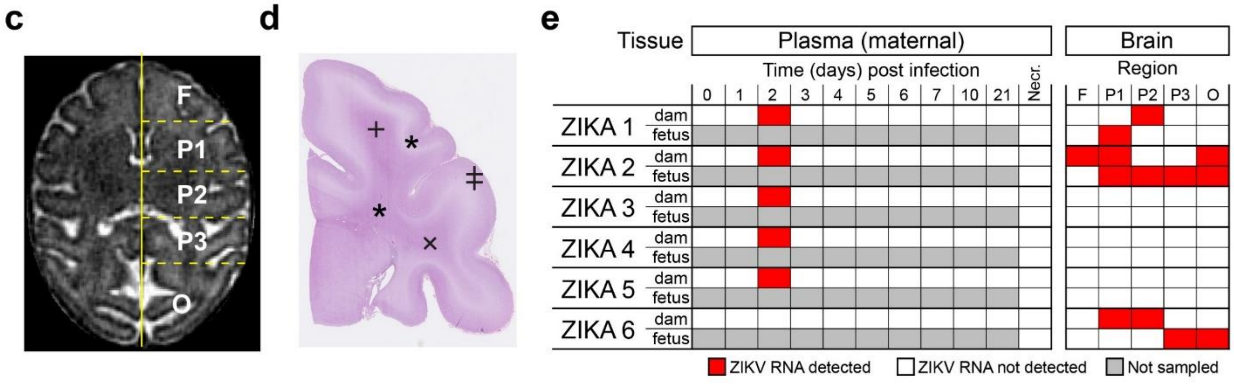

Figure S1. Maternal ZikV infection study design and NHP fetal brain sampling overview. a) A timeline of study events for ZIKA1-6 and CTL1-6 is shown with respect to the gestational age in days on the x-axis. Pregnant dams were inoculated subcutaneously with Asian-lineage ZikV clinical isolate from Cambodia (FSS13025 Cambodia 2010; GenBank no. MH368551) or American-lineage ZikV virus clinical isolate from 2015 Brazil (Brazil 2015; GenBank no. KX811222) at the gestational age indicated. Dams underwent Cesarean section at the indicated gestation age, prior to 172 days, which is the average gestational age at spontaneous delivery in the Washington National Primate Research Center (WaNPRC) colony. Magnetic resonance imaging (MRI) was performed at indicated time points; larger "MRI" icon for each animal indicates the time point analyzed in Fig 3. b) Table representing animals from which tissue was used in downstream analysis. For each assay, tissue was included for analysis only if it matched the brain location of other sections being analyzed in the same assay. This led to some brain areas for some animals not being represented in the final dataset. c) Fetal cerebrum bulk tissue dissection scheme overlaid onto an MRI of a control animal at 156 gestational days (GD) in the horizontal plane. The cerebrum was sectioned at the midline (parasagittal vertical line) and coronal sections (transverse dashed lines) collected from 5 regions, referred to as frontal (F), parietal (P1-P3), and occipital (O). Tissue from right hemisphere was used fresh for bulk RNA sequencing or preserved in formalin or 4% paraformaldehyde for immunohistochemical and electron microscopy (EM) analyses. d) The left hemisphere was submerged in formalin or 4% paraformaldehyde (PFA) and coronal slices, corresponding to P2 and O, were sectioned and embedded into paraffin for immunohistochemical (IHC) staining. The representative micrograph is a hematoxylin and eosin (H&E)-stained section from the parietotemporal region (P2) of a control animal at 155 GD. EM samples were collected from cerebral gray and white matter (*). Tissue samples used for bulk RNA sequencing were collected in the superficial grey matter (‡). Quantification of protein expression was performed in deep white matter tracts of the superior (+) or inferior (x) cortical gyri of the parietal or occipital lobe where myelin was most dense. e) Table representing the detection of ZikV RNA by TaqMan polymerase chain reaction on tissue from dam (upper row) and fetus (lower row) for each ZikV-exposed animal. Note that the three ZikV-exposed animals without detectable viral RNA in the fetus also had the longest interval between inoculation and necropsy. Left table, plasma samples; right table, brain samples.

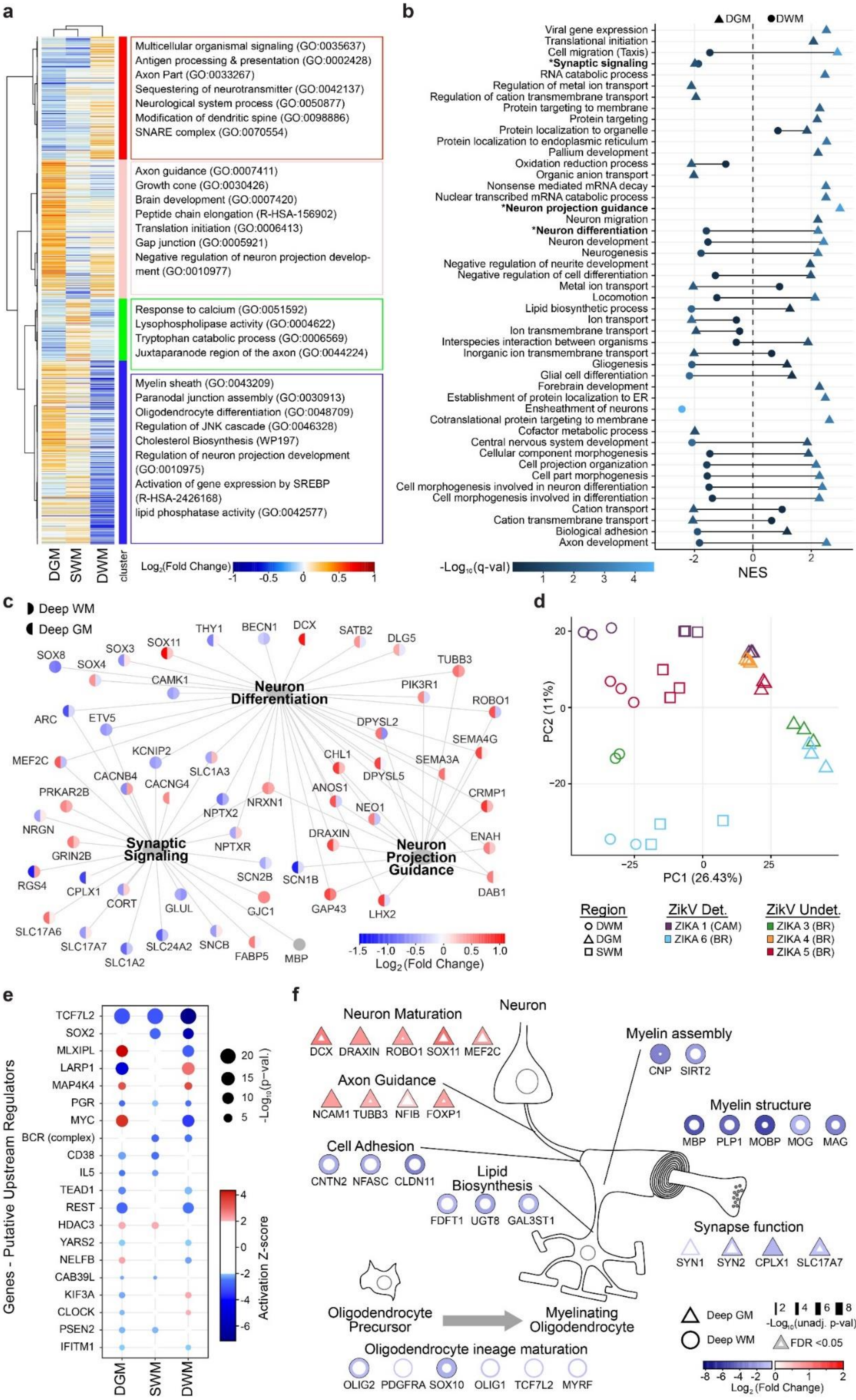

a
DGM
SWM
DWM
cluster
Multicellular organismal signaling (GO:0035637)
Antigen processing & presentation (GO:0002428)
Axon Part (GO:0033267)
Sequestering of neurotransmitter (GO:0042137)
Neurological system process (GO:0050877)
Modification of dendritic spine (GO:0098886)
SNARE complex (GO:0070554)
Axon guidance (GO:0007411)
Growth cone (GO:0030426)
Brain development (GO:0007420)
Peptide chain elongation (R-HSA-156902)
Translation initiation (GO:0006413)
Gap junction (GO:0005921)
Negative regulation of neuron projection development (GO:0010977)
Response to calcium (GO:0051592)
Lysophospholipase activity (GO:0004622)
Tryptophan catabolic process (GO:0006569)
Juxtaparanode region of the axon (GO:0044224)
Myelin sheath (GO:0043209)
Paranodal junction assembly (GO:0030913)
Oligodendrocyte differentiation (GO:0048709)
Regulation of JNK cascade (GO:0046328)
Cholesterol Biosynthesis (WP197)
Regulation of neuron projection development (GO:0010975)
Activation of gene expression by SREBP (R-HSA-2426168)
lipid phosphatase activity (GO:0042577)
Log2(Fold Change)
-1 -0.5 0 0.5 1
b
▲DGM ●DWM
Viral gene expression
Translational initiation
Cell migration (Taxis)
*Synaptic signaling
RNA catabolic process
Regulation of metal ion transport
Regulation of cation transmembrane transport
Protein targeting to membrane
Protein targeting
Protein localization to organelle
Protein localization to endoplasmic reticulum
Pallium development
Oxidation reduction process
Organic anion transport
Nonsense mediated mRNA decay
Nuclear transcribed mRNA catabolic process
*Neuron projection guidance
Neuron migration
*Neuron differentiation
Neuron development
Neurogenesis
Negative regulation of neurite development
Negative regulation of cell differentiation
Metal ion transport
Locomotion
Lipid biosynthetic process
Ion transport
Ion transmembrane transport
Interspecies interaction between organisms
Inorganic ion transmembrane transport
Gliogenesis
Glial cell differentiation
Forebrain development
Establishment of protein localization to ER
Ensheathment of neurons
Cotranslational protein targeting to membrane
Cofactor metabolic process
Central nervous system development
Cellular component morphogenesis
Cell projection organization
Cell part morphogenesis
Cell morphogenesis involved in neuron differentiation
Cell morphogenesis involved in differentiation
Cation transport
Cation transmembrane transport
Biological adhesion
Axon development
-Log10(q-val)
1 2 3 4
-2 0 2
NES
c
Deep WM
Deep GM
Neuron Differentiation
Synaptic Signaling
Neuron Projection Guidance
THY1 BECN1 DCX SATB2 DLG5 SOX11 SOX3 SOX8 SOX4 CAMK1 TUBB3 PIK3R1 ROBO1 ARC ETV5 DPYSL2 SEMA4G MEF2C KCNIP2 CHL1 SEMA3A CACNB4 SLC1A3 CACNG4 DPYSL5 PRKAR2B ANOS1 CRMP1 NRXN1 NPTX2 NEO1 NRGN DRAXIN ENAH GRIN2B NPTXR RGS4 CPLX1 SCN2B SCN1B DAB1 CORT GLUL GJC1 GAP43 LHX2 SLC17A6 SLC17A7 SLC24A2 SNCB FABP5 MBP SLC1A2
-1.5 -1.0 -0.5 0 0.5 1.0
Log2(Fold Change)
d
20 0 -20
PC2 (11%)
-25 0 25
PC1 (26.43%)
Region
DWM
DGM
SWM
ZikV Det.
ZIKA 1 (CAM)
ZIKA 6 (BR)
ZikV Undet.
ZIKA 3 (BR)
ZIKA 4 (BR)
ZIKA 5 (BR)
e
Genes - Putative Upstream Regulators
TCF7L2
SOX2
MLXIPL
LARP1
MAP4K4
PGR
MYC
BCR (complex)
CD38
IL5
TEAD1
REST
HDAC3
YARS2
NELFB
CAB39L
KIF3A
CLOCK
PSEN2
IFITM1
DGM
SWM
DWM
20 15 10 5
-Log10(p-val.)
4 2 0 -2 -4 -6
Activation Z-score
f
Neuron Maturation
DCX DRAXIN ROBO1 SOX11 MEF2C
Neuron
Myelin assembly
CNP SIRT2
Axon Guidance
NCAM1 TUBB3 NFIB FOXP1
Myelin structure
MBP PLP1 MOBP MOG MAG
Cell Adhesion
CNTN2 NFASC CLDN11
Lipid Biosynthesis
FDFT1 UGT8 GAL3ST1
Synapse function
SYN1 SYN2 CPLX1 SLC17A7
Oligodendrocyte Precursor
Myelinating Oligodendrocyte
Deep GM
Deep WM
2 4 6 8
-Log10(unadj. p-val)
FDR <0.05
Oligodendrocyte ineage maturation
OLIG2 PDGFRA SOX10 OLIG1 TCF7L2 MYRF
-8 -6 -4 -2 0 1 2
Log2 (Fold Change)

Figure S2. Spatial transcriptomic analysis of NHP fetal brain responses to ZikV infection in discrete brain regions. Digital spatial profiling (DSP) of tissue from control (n=6) and ZikV (n=6) animals. a) Left, Heatmap of average $\log_2$ fold change by ROI of 1234 differentially expressed genes identified as significant (FDR<0.05) in at least one brain region (Table S3). Orange indicates upregulated gene expression in ZikV relative to CTL. Blue indicates down-regulated gene expression in ZikV relative to CTL. Hierarchical clustering using Euclidean distance measure and ward.D2 identified 4 clusters of genes (vertical color bar). Right, functional pathway identification by Over Representation Analysis (ORA) is displayed for each cluster, according to color (Table S4). b) Plot of normalized enrichment scores (NES) for pathways identified by performing GSEA (using C5 gene ontology) in DGM (triangles) and DWM (circles) regions based on analysis of all genes detected. Color scale indicates adjusted q-values (Table S5). c) Network of 55 DE genes (|LFC|>0.5, FDR<0.05) in DGM clustered by C5 gene ontology (from Fig. S2a) representing neuron differentiation, synaptic signaling, and neuron projection guidance. Small node color represents average log-fold change (ZikV vs CTL) for each gene in DWM (left half) and DGM (right half). MBP is displayed in grey as the log fold change is outside the bounds of the color scale bar. d) Principal component analysis representing variability across samples (one point per sample; color=animal ID, shape=brain region). Animals with ZikV RNA detected at delivery (ZIKA 1 and ZIKA 6) or infected with Cambodian strain (ZIKA 1), do not show obvious separation from other animals. CAM, Cambodia; BR, Brazil. e) Dot plot of 20 significant upstream transcriptional regulators (TR) identified in at least two out of the three brain regions (DGM, SWM, and DWM) of interest using Ingenuity Pathway Analysis (IPA; Table S6). IPA Upstream Regulator analysis was performed on the 1234 DEG identified in panel a (Table S3). The dot size is proportional to significance (-log10 *p*-value); color represents the TR activation z-score (> 2 for activation or < -2 for inhibition). Genes were sorted by sum of absolute z-scores across the three ROIs, and the top three genes, *TCF7L2*, *SOX2*, and *MLXIPL*, have the most statistically significant overall z-scores. f) Functional schematic showing cellular location of genes involved in neuron health and synaptic function linked with oligodendrocyte development and myelination. Genes are colored by log fold change expression (ZikV *vs*. CTL) identified in the DSP analysis. Shape indicates the region represented in the analysis, with DGM in triangles and DWM in circles. Line thickness signifies the negative log of the unadjusted p-value, and the presence of a thin bounding black line indicates FDR<0.05.

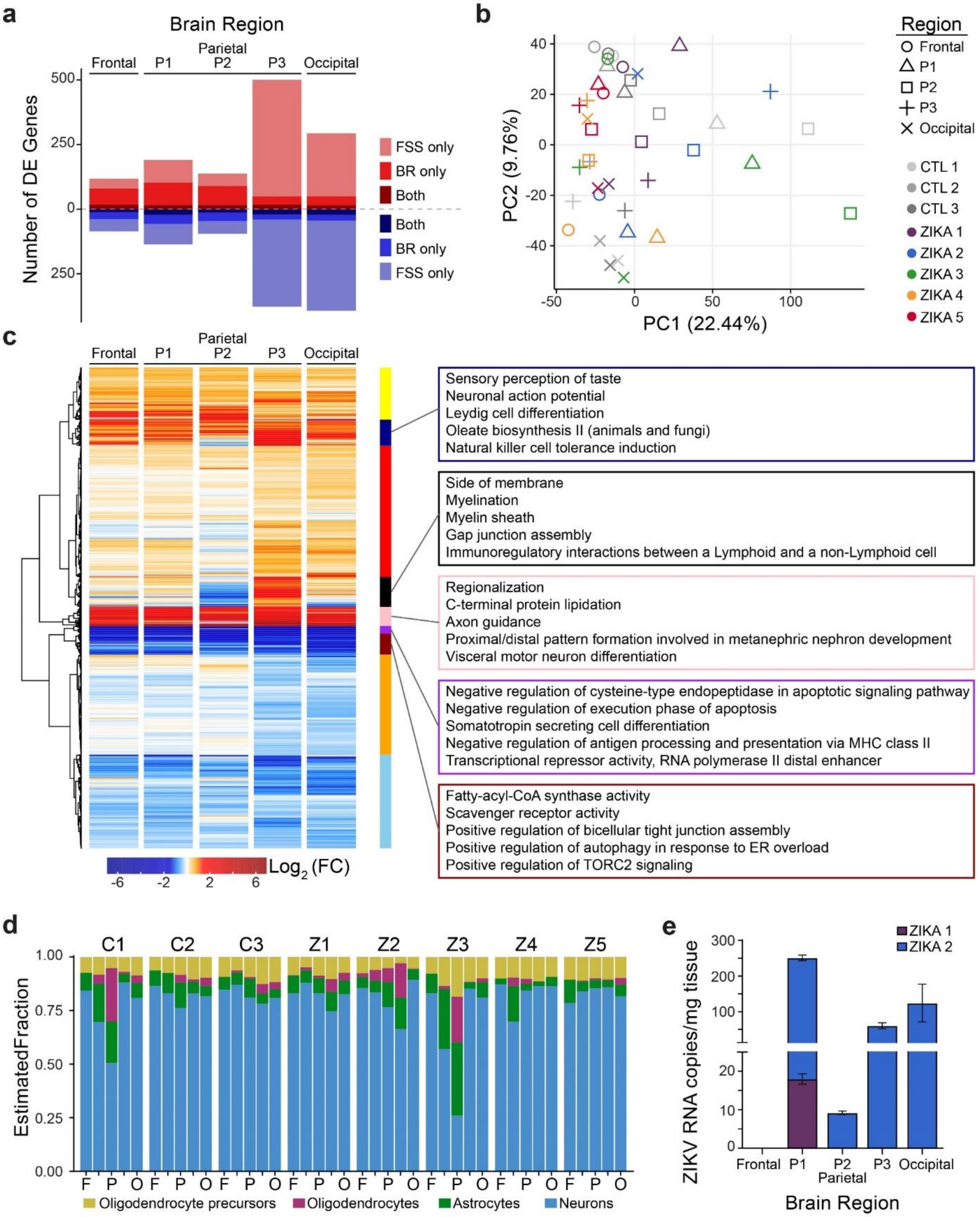
a
Brain Region
Parietal
Frontal
P1
P2
P3
Occipital
Number of DE Genes
500
250
0
250
FSS only
BR only
Both
Both
BR only
FSS only
b
Region
Frontal
P1
P2
P3
Occipital
CTL 1
CTL 2
CTL 3
ZIKA 1
ZIKA 2
ZIKA 3
ZIKA 4
ZIKA 5
PC2 (9.76%)
PC1 (22.44%)
40
20
0
-20
-40
-50
0
50
100
c
Parietal
Frontal
P1
P2
P3
Occipital
Sensory perception of taste
Neuronal action potential
Leydig cell differentiation
Oleate biosynthesis II (animals and fungi)
Natural killer cell tolerance induction
Side of membrane
Myelination
Myelin sheath
Gap junction assembly
Immunoregulatory interactions between a Lymphoid and a non-Lymphoid cell
Regionalization
C-terminal protein lipidation
Axon guidance
Proximal/distal pattern formation involved in metanephric nephron development
Visceral motor neuron differentiation
Negative regulation of cysteine-type endopeptidase in apoptotic signaling pathway
Negative regulation of execution phase of apoptosis
Somatotropin secreting cell differentiation
Negative regulation of antigen processing and presentation via MHC class II
Transcriptional repressor activity, RNA polymerase II distal enhancer
Fatty-acyl-CoA synthase activity
Scavenger receptor activity
Positive regulation of bicellular tight junction assembly
Positive regulation of autophagy in response to ER overload
Positive regulation of TORC2 signaling
Log2 (FC)
-6
-2
2
6
d
C1
C2
C3
Z1
Z2
Z3
Z4
Z5
EstimatedFraction
1.00
0.75
0.50
0.25
0.00
F P O F P O F P O F P O F P O F P O F P O F P O
Oligodendrocyte precursors
Oligodendrocytes
Astrocytes
Neurons
e
ZIKA 1
ZIKA 2
ZIKV RNA copies/mg tissue
300
200
100
20
10
0
Frontal
P1
P2
P3
Occipital
Parietal
Brain Region

Figure S3. Global RNA sequencing of ZikV and control NHP fetal superficial cortex. Bulk RNA sequencing data representing control (n=3) and ZikV (n=5) animals. a) Bar plot of the total number of differentially expressed genes (DEG) identified in comparisons of ZikV *vs.* control (CTL) for brain region-matched samples. Red indicates up-regulated genes; blue indicates down-regulated genes. The color shade represents the number of DEG in ZikV/FSS, ZikV/BR or both strains. b) Principal component analysis (PCA) of the whole transcriptome across all brain samples. Each point in the plot represents an individual sample, with brain region denoted by symbol and animal denoted by color. c) Heatmap of average log fold change (LFC) expression of 1505 DEG identified in at least one brain region (p<0.05). Hierarchical clustering was performed using Euclidean distance measure and ward.D2 and identified 9 clusters of genes (vertical color bar) (Table S7). Right, functional pathway identification by over representation analysis against gene ontology, KEGG and wikipathways pathway databases is displayed for the five clusters with the largest average differences in gene expression, according to color (Table S8). d) Stacked bar chart of relative percent abundance of individual cell types predicted in each brain sample using deconvolution analysis with CIBERSORT (Table S9). Bars are ordered left to right from frontal (F), parietal (P)1-3, and occipital (O) regions within each animal. Animal is listed across the top. Bar color denotes cell type, including oligodendrocyte precursors (gold), oligodendrocytes (purple), astrocytes (green), and neurons (blue). e) ZikV RNA was detected in the brain of two animals using a ZikV-specific qRT-PCR assay. Maternal ZIKA1 and ZIKA2 animals were inoculated with ZikV/FSS and fetal brain samples analyzed at 43 and 77 days post-inoculation, respectively (see Figure S1; note that ZikV RNA was detected in fetal brain of ZIKA6 using a different primer set and therefore not included in the quantitative representation in panel S3e). Average gestational age (±SD) of ZikV-exposed vs CTL animals in RNAseq analysis=157(±2) vs 154(±8) days; p=0.6 by t-test. Source data are provided as a Source Data file.

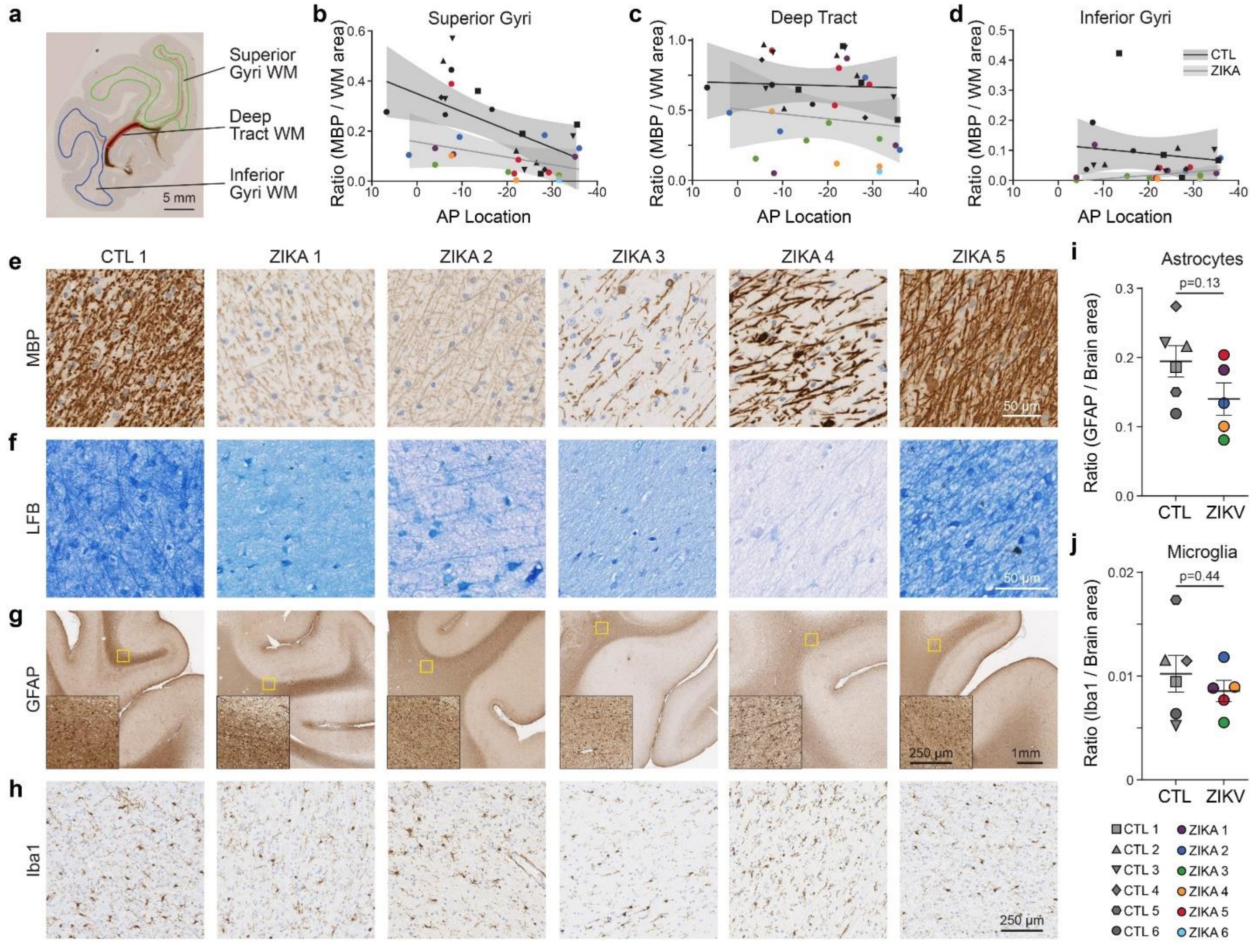
a
Superior Gyri WM
Deep Tract WM
Inferior Gyri WM
5 mm
b
Superior Gyri
c
Deep Tract
d
Inferior Gyri
CTL
ZIKA
Ratio (MBP / WM area)
AP Location
e
f
g
h
CTL 1
ZIKA 1
ZIKA 2
ZIKA 3
ZIKA 4
ZIKA 5
MBP
LFB
GFAP
Iba1
50 µm
250 µm
1mm
i
Astrocytes
p=0.13
Ratio (GFAP / Brain area)
CTL
ZIKV
j
Microglia
p=0.44
Ratio (Iba1 / Brain area)
CTL 1
CTL 2
CTL 3
CTL 4
CTL 5
CTL 6
ZIKA 1
ZIKA 2
ZIKA 3
ZIKA 4
ZIKA 5
ZIKA 6

Figure S4. Immunohistological characterization of ZikV and control NHP fetal brains. Formalin-fixed paraffin-embedded (FFPE) coronal slices of parietal cortex were used for immunohistochemical (IHC) analyses. a) section of occipital cortex (from CTL 3) stained for MBP highlighting the regions of interest (ROIs) in white matter for which MBP staining was quantified, representing subcortical WM in superior gyri (green), inferior gyri (blue) and central deep WM tracts (red). b-d) Quantification of MBP staining area in the WM from b) superior gyri, c) deep tract, and d) inferior gyri, measured as the ratio of area occupied by chromogen divided by the total area of the ROI. AP Location refers to distance from the anterior commissure along the rostro-caudal axis (negative values are more caudal) referenced to the adult Macaque Scalable Brain Atlas (see Methods). Each point represents an individual section; for control, n=6 animals, 17 tissue blocks; for ZikV, n=6 animals, 19 tissue blocks. Black line, linear regression of CTL points; grey line, linear regression of ZikV points; shaded areas, 95% confidence interval. e) High-magnification micrographs of myelin basic protein (MBP) IHC from central areas of deep white matter (DWM) containing dense fiber tracts, demonstrating reduced intensity of MBP signal, as well as decreased density of MBP-labeled fibers in ZikV virus cohort animals. f) Luxol fast blue combined with periodic acid-Schiff (LFB-PAS) staining was used to detect compact myelin. g) IHC for astrocyte marker, glial fibrillary acidic protein (GFAP), with insets demonstrating high-magnification areas of the DWM tracts. h) IHC for the microglial marker, allograft inflammatory factor 1 (AIF-1/Iba), obtained from the DWM tracts. The regions selected are approximately the same location as the inset images in panel c. i-j) Quantification of i) GFAP and j) Iba1 signal throughout the parietal cortex expressed as the ratio of area occupied by the chromogen divided by the total area of brain tissue present within the section. A single section per animal (n=6 CTL, n=5 ZikV) was quantified. Points in the plots represent individual animals, with bars indicating mean and standard error mean (SEM). Differences between ZikV and CTL animals were not significant for either GFAP or Iba1 by t-test with Welch's correction. Average gestational age (±SD) of ZikV-exposed vs CTL animals in IHC analysis=152(±2) *vs.* 154(±8) days; p=0.47 by t-test. Source data are provided as a Source Data file.

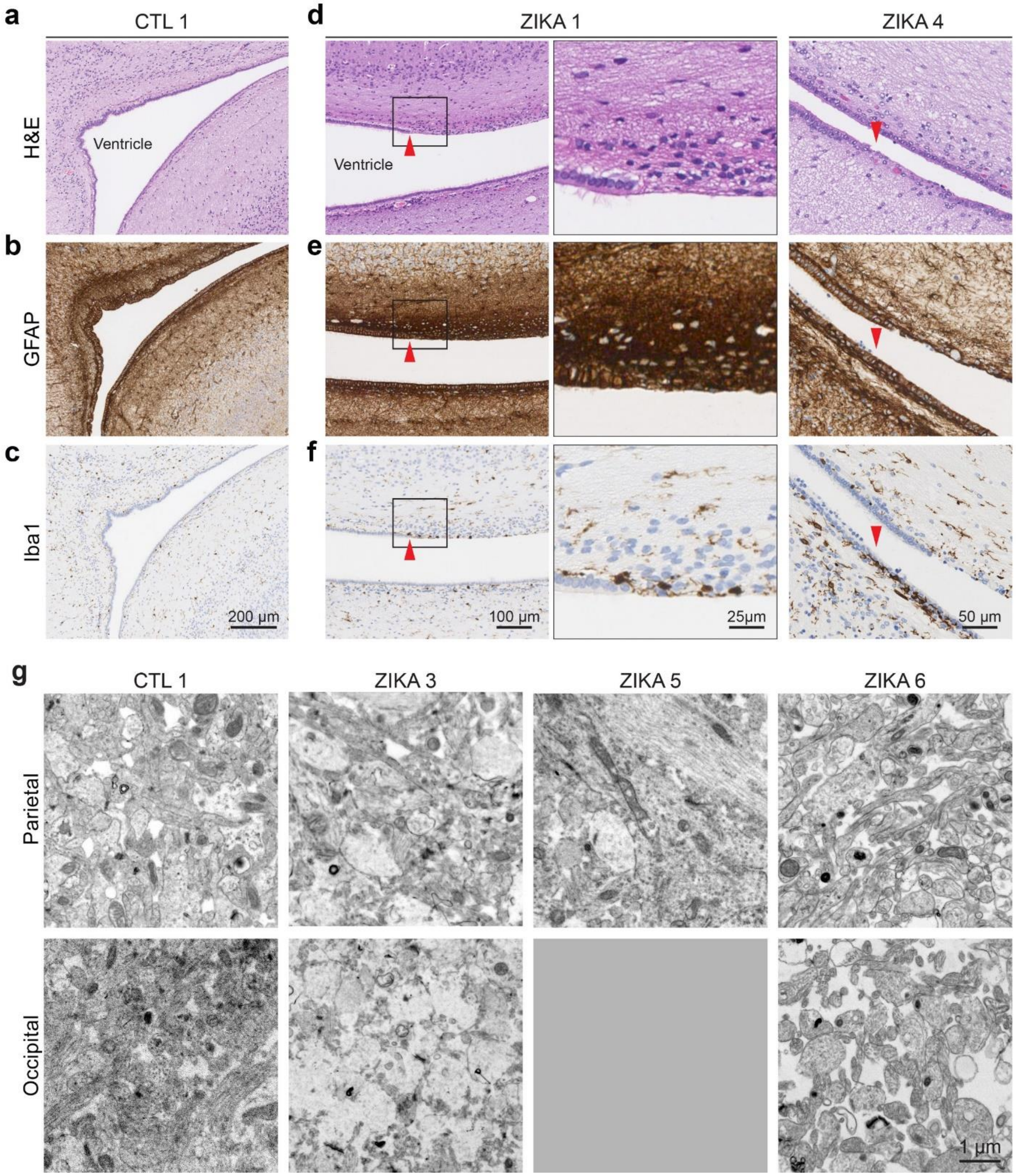
a
CTL 1
d
ZIKA 1
ZIKA 4
H&E
Ventricle
Ventricle
b
e
GFAP
c
f
Iba1
200 µm
100 µm
25µm
50 µm
g
CTL 1
ZIKA 3
ZIKA 5
ZIKA 6
Parietal
Occipital
1 µm

Figure S5. Histopathologic changes in the periventricular region of the occipital cortex lesion of ZikV and control NHP fetal brain. Formalin-fixed paraffin-embedded (FFPE) coronal section of occipital cortex was used for hematoxylin and eosin (H&E) staining and immunohistochemical (IHC) analyses. a-f) H&E, glial fibrillary acidic protein (GFAP) and allograft inflammatory factor 1 (AIF-1/Iba1) stained sections of the lateral ventricle and ependymal lining of CTL1 (a-c) and similarly stained sections from the same region in ZikV-exposed animals (d-f). Inset in the right column of images from ZIKA 1 shows high-magnification images of the area marked by the black rectangle. Red arrowheads indicate a transition point where the ciliated ependymal lining is replaced by an area with multiple vacuoles and increased cellularity. This area also had increased GFAP staining intensity and a higher density of Iba1-positive microglia. g) representative EM images of brain tissue collected from grey matter in the posterior parietal lobe (top) and occipital lobe (bottom) for CTL (left) and ZikV animals (right). Grey box for occipital section from ZIKA 5 indicates that tissue was not available from this region for this animal. Scale, shown in panel g, is identical across primary images.

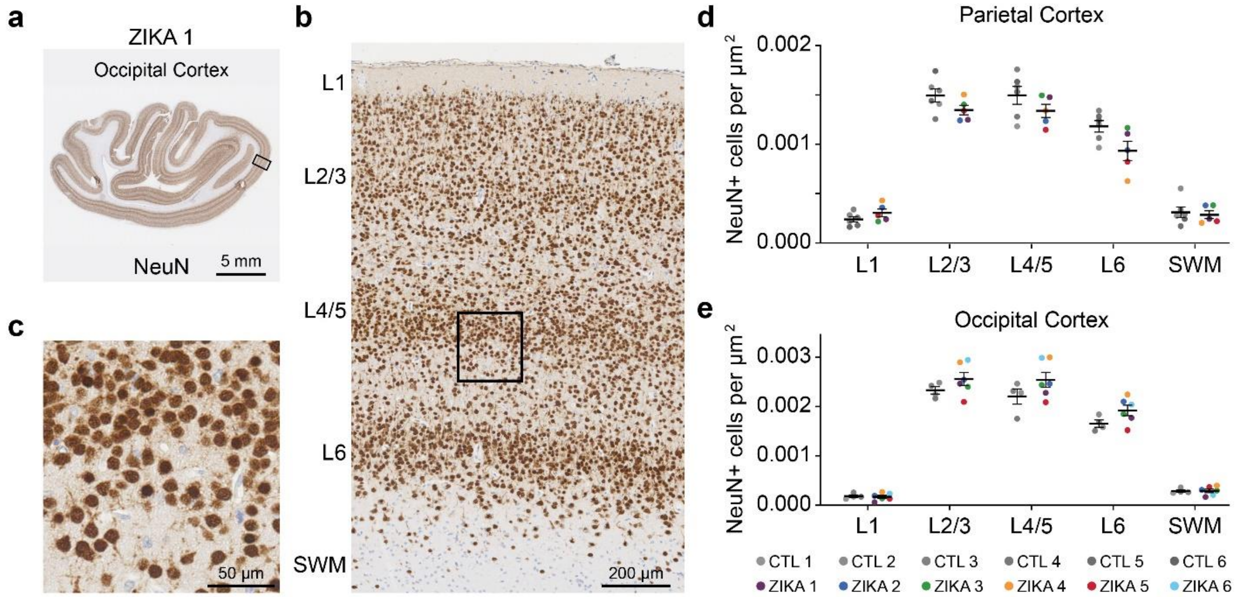

Figure S6. Neuron density by cortical layer in parietal and occipital cortex of ZikV and control NHP fetal brains. Formalin-fixed paraffin-embedded (FFPE) coronal sections of parietal and occipital cortex were used for immunohistochemical (IHC) analyses. a-c) Neuronal nuclear protein (NeuN)-stained coronal section of occipital cortex in a ZikV virus cohort animal (ZIKA 1). b, cortical layers 1-6 (L1-L6) and subcortical white matter (SWM) are indicated. c) inset shown by the black rectangle in panel b of the neuronal bodies. d-e) Semi-automated quantification of neuron density in the d) parietal and e) occipital cortex. Regions of interest (ROI) delineating individual cortical layers (L1, L2/3, L4/5, L6) and SWM were manually drawn, as in panel b, and NeuN+ cells were counted within each ROI using automated Visiopharm software. Points in the plots represent individual animals (n=6 CTL, n=6 ZikV), from which three independent areas containing all cortical layers were quantified and averaged together, with bars indicating mean and standard error mean (SEM). Source data are provided as a Source Data file.

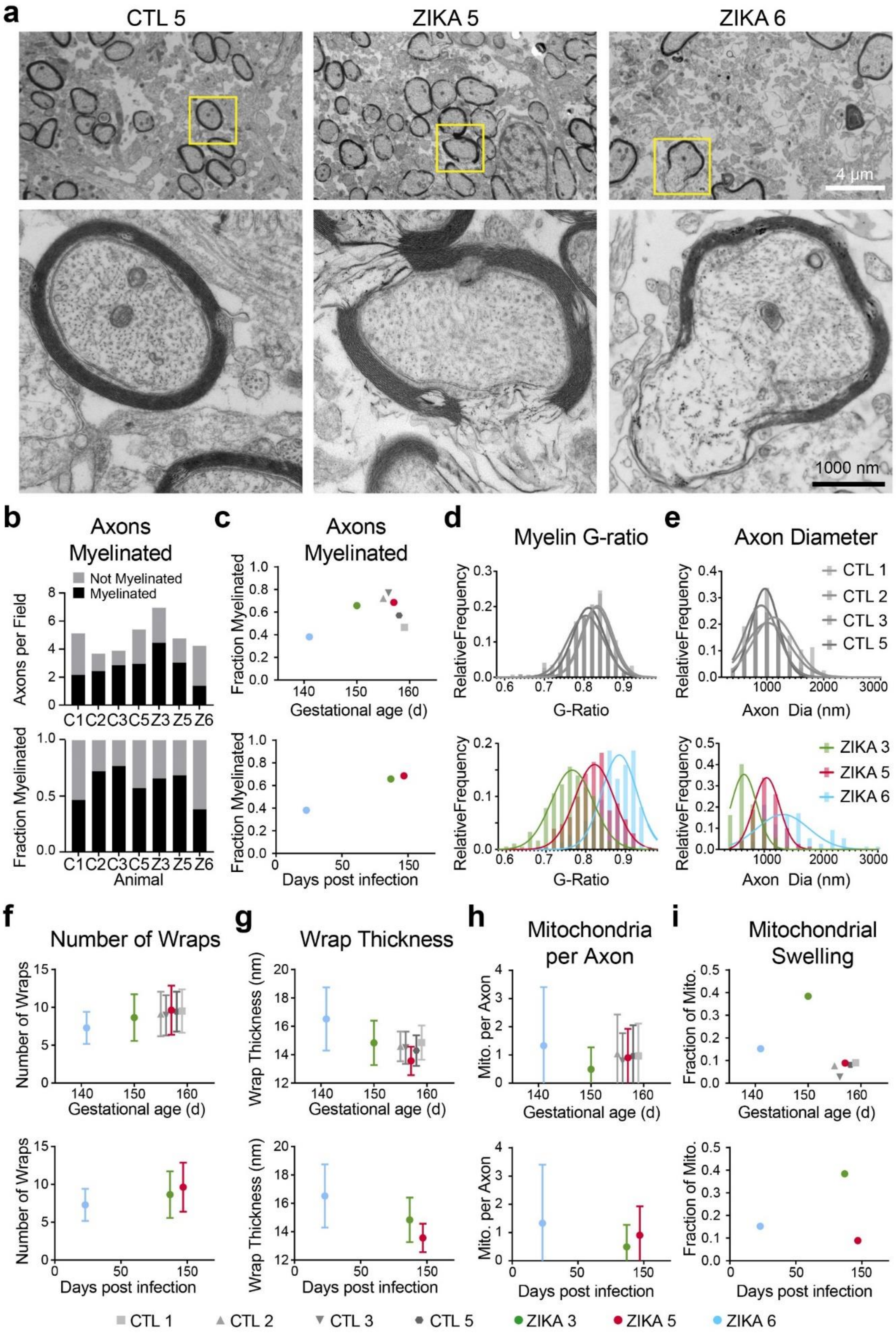
a
CTL 5
ZIKA 5
ZIKA 6
4 µm
1000 nm
b
Axons Myelinated
Not Myelinated
Myelinated
Axons per Field
Fraction Myelinated
C1 C2 C3 C5 Z3 Z5 Z6
Animal
c
Axons Myelinated
Fraction Myelinated
Gestational age (d)
Days post infection
d
Myelin G-ratio
RelativeFrequency
G-Ratio
e
Axon Diameter
RelativeFrequency
Axon Dia (nm)
CTL 1
CTL 2
CTL 3
CTL 5
ZIKA 3
ZIKA 5
ZIKA 6
f
Number of Wraps
Number of Wraps
Gestational age (d)
Days post infection
g
Wrap Thickness
Wrap Thickness (nm)
Gestational age (d)
Days post infection
h
Mitochondria per Axon
Mito. per Axon
Gestational age (d)
Days post infection
i
Mitochondrial Swelling
Fraction of Mito.
Gestational age (d)
Days post infection
CTL 1
CTL 2
CTL 3
CTL 5
ZIKA 3
ZIKA 5
ZIKA 6

Figure S7. Electron microscopy analysis of axon and myelin features in deep white matter of ZikV and control NHP fetal brains. a) top row, representative EM images of deep white matter from CTL 5, ZIKA 5 and ZIKA 6 animals. Inset in the bottom row shows high-magnification (40,000x) images of the area marked by the yellow rectangle, which are representative of the images used for quantification. Scale, shown in panel a, is identical across primary images. b) quantification of the number (top) or fraction (bottom) of large-diameter (>250 nm) axons with mature myelin sheaths. c) analysis of the fraction of axons myelinated according to gestational age (top row) or days post-ZikV inoculation (bottom row). d-e) Histograms representing d) myelin g-ratio and e) axon diameter across all analyzed axons for each animal in control (top) and ZikV-exposed (bottom) groups. f-i) Quantification of myelin features (f-g) and mitochondrial features (h-i) per axon in control and ZikV-exposed animals, plotted relative to gestational age (top row) or days post-ZikV inoculation (bottom row). Each colored point (graphs c, f-h) represents the average of all measurements for a single animal; error bars, when shown, indicate the mean and SD of measurements across axons for each animal. 617 axons (n=4 CTL); 501 axons (n= 3 ZikV). Source data are provided as a Source Data file.

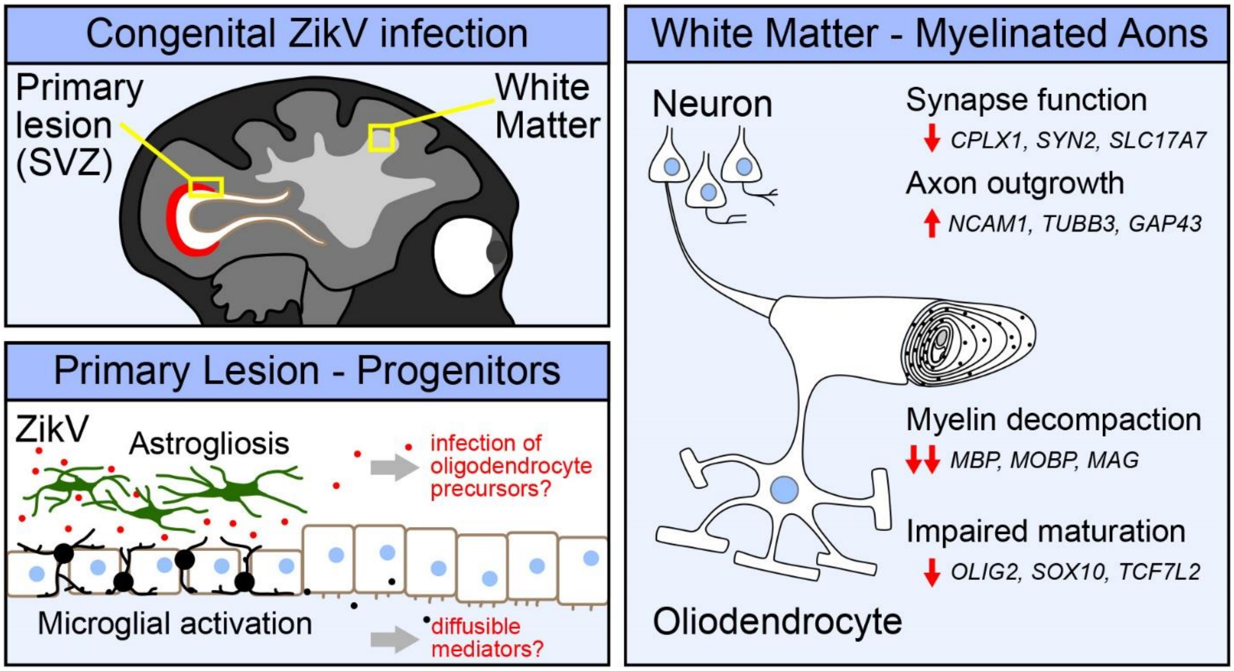

Figure S8. Graphical abstract representing putative mechanisms for ZikV-induced myelin decompaction, including altered oligodendrocyte maturation, and neuronal function. In a nonhuman primate model of non-microcephalic congenital Zika syndrome, we identified a primary lesion affecting the posterior periventricular area corresponding to the neural progenitor cell niche in the subventricular zone (SVZ), in which focal astrogliosis and reactive microglia were prominent features of pathology. In parietal cortex distal to this site, we found extensive perturbations of myelin with decompaction of the myelin sheath and loss of myelin basic protein expression. Spatially resolved transcriptional analysis identified ZikV-related changes in gene networks in the white matter with downregulation of oligodendrocyte functional genes, while in the grey matter genes for axon outgrowth were upregulated. We hypothesize congenital ZikV infection perturbs core oligodendrocyte transcriptional programs, either via direct infection of oligodendrocyte precursors or via diffusible mediators, leading to downregulation of genes necessary for maturation of oligodendrocyte lineage cells and strong downregulation of genes for myelin structural proteins. Loss of structural proteins such as myelin basic protein (MBP) leads to decompaction of myelin. In neurons, ZikV leads to loss of synaptic input, either because of decreased neurogenesis or because of dysfunctional electrical activity due to demyelination. In response, neurons increase expression of genes for axon outgrowth in order to make contact with new synaptic partners.
