# Supplementary material for: Disruption of myelin structure and oligodendrocyte maturation in a macaque model of congenital Zika infection
Source: Nat Commun. 2024 Jun 18;15:5173. doi: 10.1038/s41467-024-49524-2 (PMC11189406; doi:10.1038/s41467-024-49524-2)
Supplement: Supplementary file 3 — Description of Additional Supplementary Files [file 41467_2024_49524_MOESM3_ESM.docx]

**Supplementary Data 1. DSP differentially expressed gene matrix from Fig. S2a.**

Log fold change and significance were calculated from linear mixed model using GeoMx Tools R package (see methods), resulting in an unadjusted two-sided p-value for each gene comparison and a false-discovery rate (FDR), calculated from the p-value using the Benjamini-Hochberg method.

**Supplementary Data 2. Over representation gene set analysis from the heatmap shown in Fig. S2a**

False discovery rate calculated from the p-value using the Benjamini-Hochberg method.

**Supplementary Data 3.** **GSEA results from DE genes by ROI, shown in Fig. S2b**

Normalized enrichment score calculated using gene set enrichment analysis. False discovery rate calculated from the p-value using the Benjamini-Hochberg method.

**Supplementary Data 4. Ingenuity Pathway Upstream Regulator Analysis shown in Fig. S2c**

Enrichment is reported as unadjusted z-score as calculated by Ingenuity Pathway Analysis software (Qiagen, Inc.), which uses the Fisher's Exact test to estimate a right-tailed p-value.

**Supplementary Data 5. Bulk RNA-seq differential gene expression matrix from Fig. S3c**

Log fold change values were calculated with adjusted p-values calculated using the Benjamini Hochberg method.

**Supplementary Data 6. Over Representation gene set analysis from the heatmap shown in Fig. S3c.** False discovery rate calculated from the p-value using the Benjamini-Hochberg method.

**Supplementary Data 7. CIBERSORT analysis of bulk RNA-seq data shown in Fig. S3d**

Cibersort estimates the percentage of each cell type in each sample. Estimates of statistical significance are not performed^35^.
