# Supplementary material for: Disruption of myelin structure and oligodendrocyte maturation in a macaque model of congenital Zika infection
Source: Nat Commun. 2024 Jun 18;15:5173. doi: 10.1038/s41467-024-49524-2 (PMC11189406; doi:10.1038/s41467-024-49524-2)
Supplement: Supplementary file 11 — Reporting Summary [file 41467_2024_49524_MOESM11_ESM.pdf]

nature portfolio

Corresponding author(s): Jennifer Tisoncik-Go, Caleb Stokes, Michael Gale, Jr.

Last updated by author(s): May 14, 2024

Reporting Summary

Nature Portfolio wishes to improve the reproducibility of the work that we publish. This form provides structure for consistency and transparency in reporting. For further information on Nature Portfolio policies, see our Editorial Policies and the Editorial Policy Checklist.

Statistics

For all statistical analyses, confirm that the following items are present in the figure legend, table legend, main text, or Methods section.

n/a	Confirmed	
☐	☒	The exact sample size (*n*) for each experimental group/condition, given as a discrete number and unit of measurement
☐	☒	A statement on whether measurements were taken from distinct samples or whether the same sample was measured repeatedly
☐	☒	The statistical test(s) used AND whether they are one- or two-sided<br>*Only common tests should be described solely by name; describe more complex techniques in the Methods section.*
☐	☒	A description of all covariates tested
☐	☒	A description of any assumptions or corrections, such as tests of normality and adjustment for multiple comparisons
☐	☒	A full description of the statistical parameters including central tendency (e.g. means) or other basic estimates (e.g. regression coefficient) AND variation (e.g. standard deviation) or associated estimates of uncertainty (e.g. confidence intervals)
☐	☒	For null hypothesis testing, the test statistic (e.g. *F*, *t*, *r*) with confidence intervals, effect sizes, degrees of freedom and *P* value noted<br>*Give P values as exact values whenever suitable.*
☒	☐	For Bayesian analysis, information on the choice of priors and Markov chain Monte Carlo settings
☐	☒	For hierarchical and complex designs, identification of the appropriate level for tests and full reporting of outcomes
☒	☐	Estimates of effect sizes (e.g. Cohen's *d*, Pearson's *r*), indicating how they were calculated

Our web collection on statistics for biologists contains articles on many of the points above.

Software and code

Policy information about availability of computer code

Data collection	GeoMx v2.5 (Spatial transcriptomics); NanoZoomer v2.5, (Immunohistochemistry, Histology)
Data analysis	The R codes applied to transcriptomic analyses can be accessed at DOI 10.5281/zenodo.10998903 [https://zenodo.org/records/10998903] and DOI 10.5281/zenodo.10998921 [https://zenodo.org/records/10998921]. ImageJ v1.52 (Electron Microscopy); VisioPharm v2023.01 (Immunohistochemistry, Histology); GraphPad Prism v9-10; and Microsoft Excel v2016 software were also used for data analysis.

For manuscripts utilizing custom algorithms or software that are central to the research but not yet described in published literature, software must be made available to editors and reviewers. We strongly encourage code deposition in a community repository (e.g. GitHub). See the Nature Portfolio guidelines for submitting code & software for further information.

Data

Policy information about availability of data

All manuscripts must include a data availability statement. This statement should provide the following information, where applicable:

- Accession codes, unique identifiers, or web links for publicly available datasets
- A description of any restrictions on data availability
- For clinical datasets or third party data, please ensure that the statement adheres to our policy

Transcriptomics data sets are available in the NCBI Gene Expression Omnibus (GEO) under accession number GSE226401 (bulk RNA-seq) andGSE22753 (spatial transcriptomics).

Research involving human participants, their data, or biological material

Policy information about studies with human participants or human data. See also policy information about sex, gender (identity/presentation), and sexual orientation and race, ethnicity and racism.

Reporting on sex and gender	N/A
Reporting on race, ethnicity, or other socially relevant groupings	N/A
Population characteristics	N/A
Recruitment	N/A
Ethics oversight	N/A

Note that full information on the approval of the study protocol must also be provided in the manuscript.

Field-specific reporting

Please select the one below that is the best fit for your research. If you are not sure, read the appropriate sections before making your selection.

☒ Life sciences ☐ Behavioural & social sciences ☐ Ecological, evolutionary & environmental sciences

For a reference copy of the document with all sections, see nature.com/documents/nr-reporting-summary-flat.pdf

Life sciences study design

All studies must disclose on these points even when the disclosure is negative.

Sample size	Not applicable. Sample size was not determined a priori. The modest sample size was constrained by ethical use of nonhuman primates and expense of the study.
Data exclusions	None.
Replication	Experimental findings were reliably reproduced in 4-6 Zika-exposed animals (variable according to experiment) and were absent in all controls.
Randomization	Randomization was not performed given the logistical constraints of a nonhuman primate study of maternal-fetal infection. Covariates including sex, gestational age at inoculation, gestational age at delivery, and virus strain used for inoculation were analyzed post-hoc.
Blinding	Investigators were blinded as to case and control status for data acquisition of spatial transcriptomic data, and for quantitative analysis of electron microscopy, histology, and immunohistochemistry data. This is described in the methods section.

Reporting for specific materials, systems and methods

We require information from authors about some types of materials, experimental systems and methods used in many studies. Here, indicate whether each material, system or method listed is relevant to your study. If you are not sure if a list item applies to your research, read the appropriate section before selecting a response.

Materials & experimental systems

n/a	Involved in the study	
☐	☒	Antibodies
☐	☒	Eukaryotic cell lines
☒	☐	Palaeontology and archaeology
☐	☒	Animals and other organisms
☒	☐	Clinical data
☒	☐	Dual use research of concern
☒	☐	Plants

Methods

n/a	Involved in the study	
☒	☐	ChIP-seq
☒	☐	Flow cytometry
☐	☒	MRI-based neuroimaging

Antibodies

Antibodies used	See Supplemental Table 10 for the antibody used for either IHC or GeoMx DSP analyses. Dako: GFAP (#Z0224); Novus Biologicals:

Antibodies used	AIF-1/Iba1 (#MBP2-19019), GFAP (Clone GA-5 #NBP-33184DL594); Millipore: Olig2 (#AB9610), NeuN (Clone A60, #MAB377); Abcam: MBP (Clone 12, #ab7349), RBFOX3 (Clone EPR12763, #ab190195); ThermoFisher: STYO 83 (#S11364), Goat anti-Rb AF647 (#A27040).
Validation	Primary antibodies listed as cross-reactive with human and macaque species were selected for IHC and GeoMx DSP testing. All antibodies were first validated with pigtail macaque brain tissues.

Eukaryotic cell lines

Policy information about cell lines and Sex and Gender in Research

Cell line source(s)	Vero cells were obtained from the World Health Organization. C6/36 Aedes albopictus cells were obtained from ATCC.
Authentication	Neither the Vero cell line nor the C6/36 cell line used in this study were authenticated.
Mycoplasma contamination	Vero cells and C6/36 cell lines were tested and negative for mycoplasma contamination using the MycoAlert Mycoplasma Test Kit from Lonza (#LT07-318).
Commonly misidentified lines (See ICLAC register)	No commonly misidentified cell lines were used in this study.

Animals and other research organisms

Policy information about studies involving animals; ARRIVE guidelines recommended for reporting animal research, and Sex and Gender in Research

Laboratory animals	There were a total of twelve, healthy pregnant pigtail macaques (Macaca nemestrina). Control cohort1: 6 pregnant females, 5-14 years; Zika cohort: 6 pregnant females, 9-12.8 years.
Wild animals	No wild animals were used in these studies.
Reporting on sex	All fetuses except for 2 animals (one from each experimental group) were female. We were unable to control for fetal sex in the study design and as there was a female bias, we did not consider sex as a covariate in the analyses of fetal samples.
Field-collected samples	No field-collected samples were used in these studies.
Ethics oversight	The nonhuman primate experiments were carried out in strict accordance with the recommendations in the Guide for the Care and Use of Laboratory Animals of the National Research Council and the Weatherall report, “The use of non-human primates in research”. The Institutional Animal Care and Use Committee of the University of Washington approved the study (Permit Number: 4165-02).

Note that full information on the approval of the study protocol must also be provided in the manuscript.

Plants

Seed stocks	N/A
Novel plant genotypes	N/A
Authentication	N/A

Magnetic resonance imaging

Experimental design

Design type	The experimental design is listed in the methods.
Design specifications	Structural Imaging only
Behavioral performance measures	N/A

Acquisition

Imaging type(s)	Structural Imaging only
Field strength	3T
Sequence & imaging parameters	A 2D single-shot, half-Fourier turbo spin echo multi-slice sequence (HASTE) was used to acquire T2-weighted images with the following parameters: TR/TE = 2200/160 ms, SENSE acceleration factor = 2, TSE factor = 100, along with fat suppression, and fold-over artifact suppression. Multiple contiguous 2D image stacks, with in-plane resolutions of 0.5 × 0.5 mm and thicknesses of 2 mm, were acquired along the axial, sagittal, and coronal axes of the fetal brain to facilitate the reconstruction of a 3D volume with isotropic spatial resolution.
Area of acquisition	Whole (fetal) brain

Diffusion MRI ☐ Used ☒ Not used

Preprocessing

Preprocessing software	N/a
Normalization	N/A
Normalization template	N/A
Noise and artifact removal	N/A
Volume censoring	N/A

Statistical modeling & inference

Model type and settings	N/A
Effect(s) tested	N/A

Specify type of analysis: ☒ Whole brain ☐ ROI-based ☐ Both

Statistic type for inference (See Eklund et al. 2016)	N/A
Correction	N/A

Models & analysis

n/a	Involved in the study
☒	☐ Functional and/or effective connectivity
☒	☐ Graph analysis
☒	☐ Multivariate modeling or predictive analysis
